# Supplementary material for: Carbonic Anhydrase IX-Targeted α-Radionuclide Therapy with 225Ac Inhibits Tumor Growth in a Renal Cell Carcinoma Model
Source: Pharmaceuticals (Basel). 2022 May 2;15(5):570. doi: 10.3390/ph15050570 (PMC9142961; doi:10.3390/ph15050570)
Supplement: Supplementary file 1 [file pharmaceuticals-15-00570-s001.zip › pharmaceuticals-1638236-supplementary.pdf]

Supplementary Material

Carbonic anhydrase IX-targeted α-radionuclide therapy with 225Ac inhibits tumor growth in a renal cell carcinoma model

Robin I.J. Merkx [1,2*], Mark Rijpkema[1], Gerben M. Franssen[1], Annemarie Kip[1], Bart Smeets[3], Alfred Morgenstern[4], Frank Bruchertseifer[4], Eddie Yan[5], Michael P. Wheatcroft[5], Egbert Oosterwijk[2], Peter F.A. Mulders[2], Sandra Heskamp[1]

[1]Department of Medical Imaging: Nuclear Medicine, Radboudumc, Nijmegen, The Netherlands [2]Department of Urology, Radboudumc, Nijmegen, The Netherlands
[3]Department of Pathology, Radboudumc, Nijmegen, The Netherlands
[4]European Commission, Joint Research Centre (JRC), Karlsruhe, Germany
[5]Telix Pharmaceuticals Limited, Melbourne, Australia

Correspondence: Robin.Merkx@radboudumc.nl;

Table S1.

The weight, the absolute uptake, and relative uptake over time of the spleen in mice treated with 50 kBq ^{225}Ac-hG250.

Mouse	Day 1			Day 3			Day 7		
	Weight (g)	Absolute Uptake (%ID)	Relative Uptake (%ID/g)	Weight (g)	Absolute Uptake (%ID)	Relative Uptake (%ID/g)	Weight (g)	Absolute Uptake (%ID)	Relative Uptake (%ID/g)
1	0.0716	0.390	5.450	0.0266	0.236	8.864	0.0138	0.189	13.687
2	0.0729	0.443	6.083	0.031	0.311	10.024	0.0178	0.315	17.679
3	0.0782	0.484	6.185	0.0475	0.305	6.415	0.0147	0.268	18.218
4	0.0699	0.414	5.928	0.0413	0.421	10.198	0.1703	0.155	0.913
5	0.0639	0.334	5.228	0.0573	0.546	9.524	0.0182	0.260	14.273

Table S2.

The weight, the absolute uptake, and relative uptake over time of the spleen in mice treated with 0.2 MBq ^{177}Lu-hG250.

Mouse		Day 1				Day 3				Day 7		
		Weight (g)	Absolute Uptake (%ID)	Relative Uptake (%ID/g)		Weight (g)	Absolute Uptake (%ID)	Relative Uptake (%ID/g)		Weight (g)	Absolute Uptake (%ID)	Relative Uptake (%ID/g)
1		0.0802	0.502	6.259		0.1198	0.453	3.784		0.1571	0.399	2.538
2		0.1038	0.513	4.947		0.1071	0.499	4.655		0.1276	0.332	2.601
3		0.0822	0.360	4.374		0.0798	0.338	4.235		0.2067	0.430	2.078
4		0.1586	0.405	2.556		0.2136	1.044	4.889		0.1112	0.310	2.789
5		0.1135	0.333	2.930		0.1091	0.408	3.744		0.1618	0.490	3.026

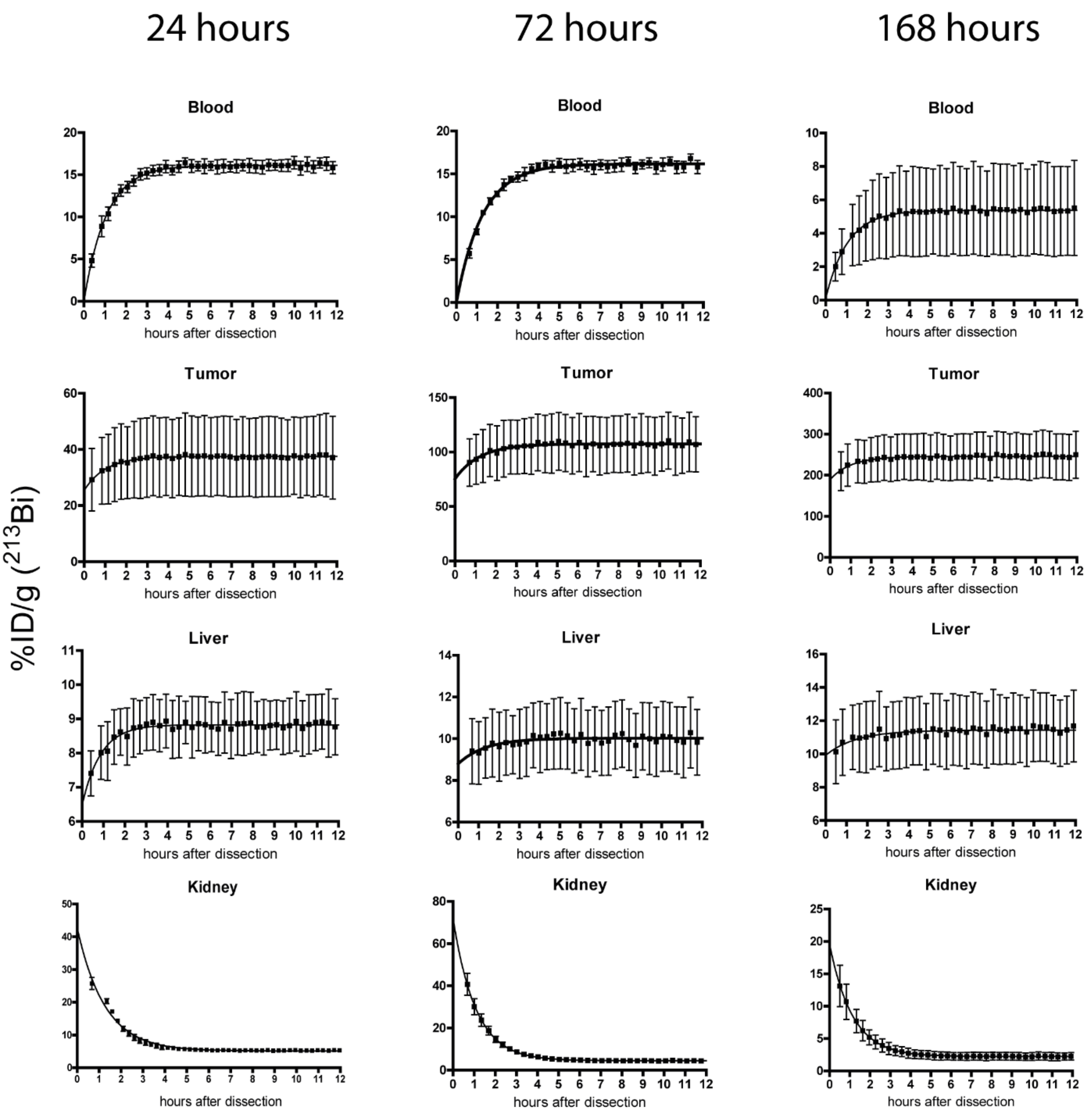

Figure S1. Continuous measurement of ^{213}Bi in organs of interest (blood, tumor, liver, and kidney) at three time points post-administration. All data points are based on the mean ± SD on that time point (n=5). The curve was obtained by applying a one-phase decay fit in all groups. The Y-axis on all graphs represents %ID/g of ^{213}Bi.

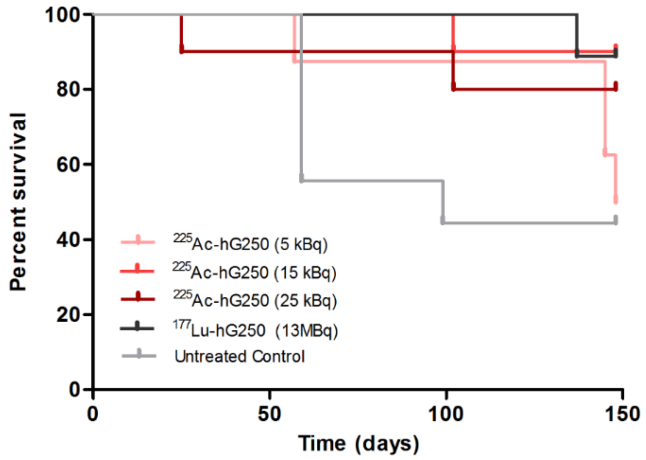

Figure S2. Survival curves of SK-RC-52 tumor-bearing mice, either untreated or treated with different activity doses of ^{225}Ac-hG250 or ^{177}Lu-hG250.

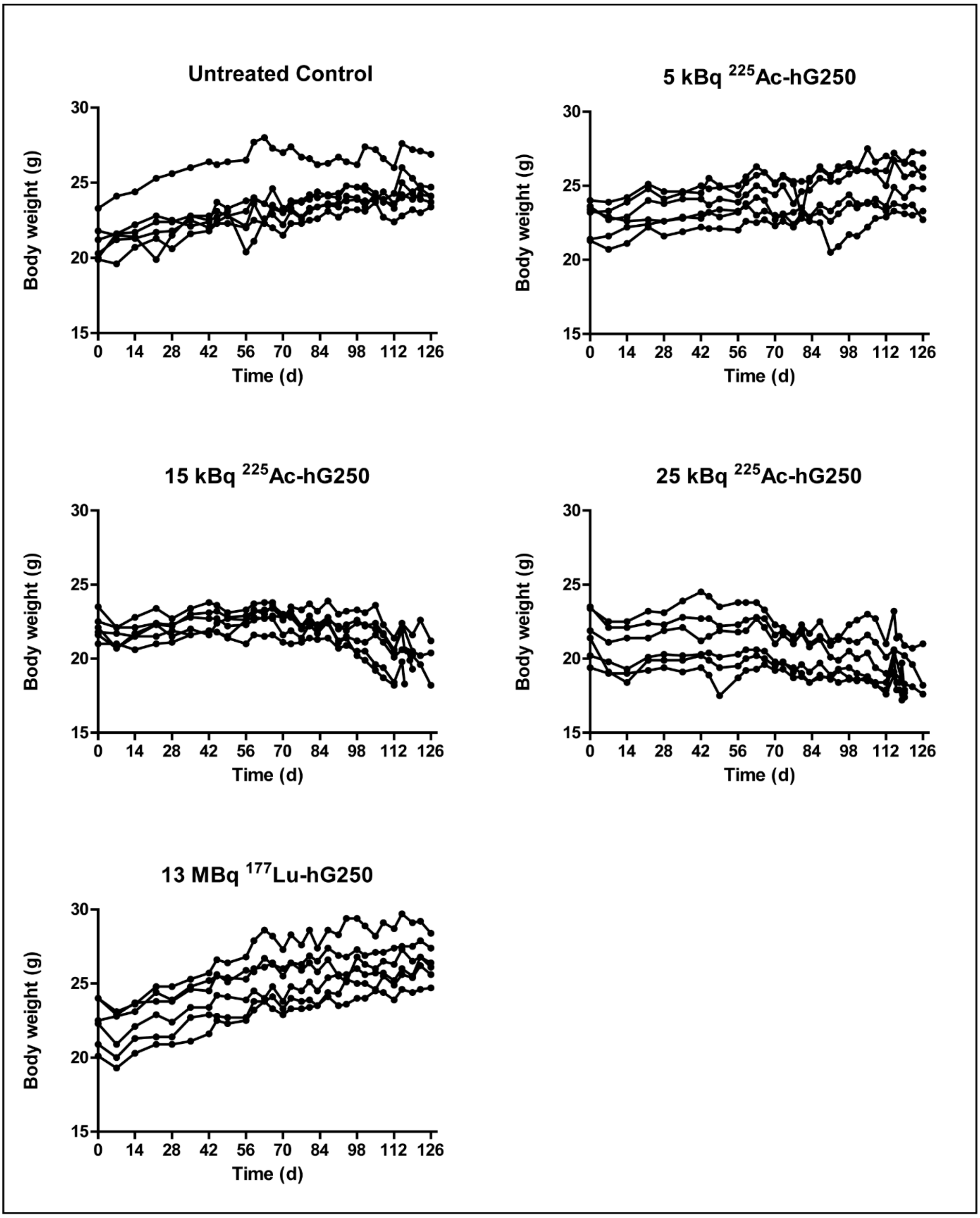

Figure S3. Absolute body weight over time in non-tumor-bearing mice either untreated or treated with different doses of ^{225}Ac-hG250 or ^{177}Lu-hG250.

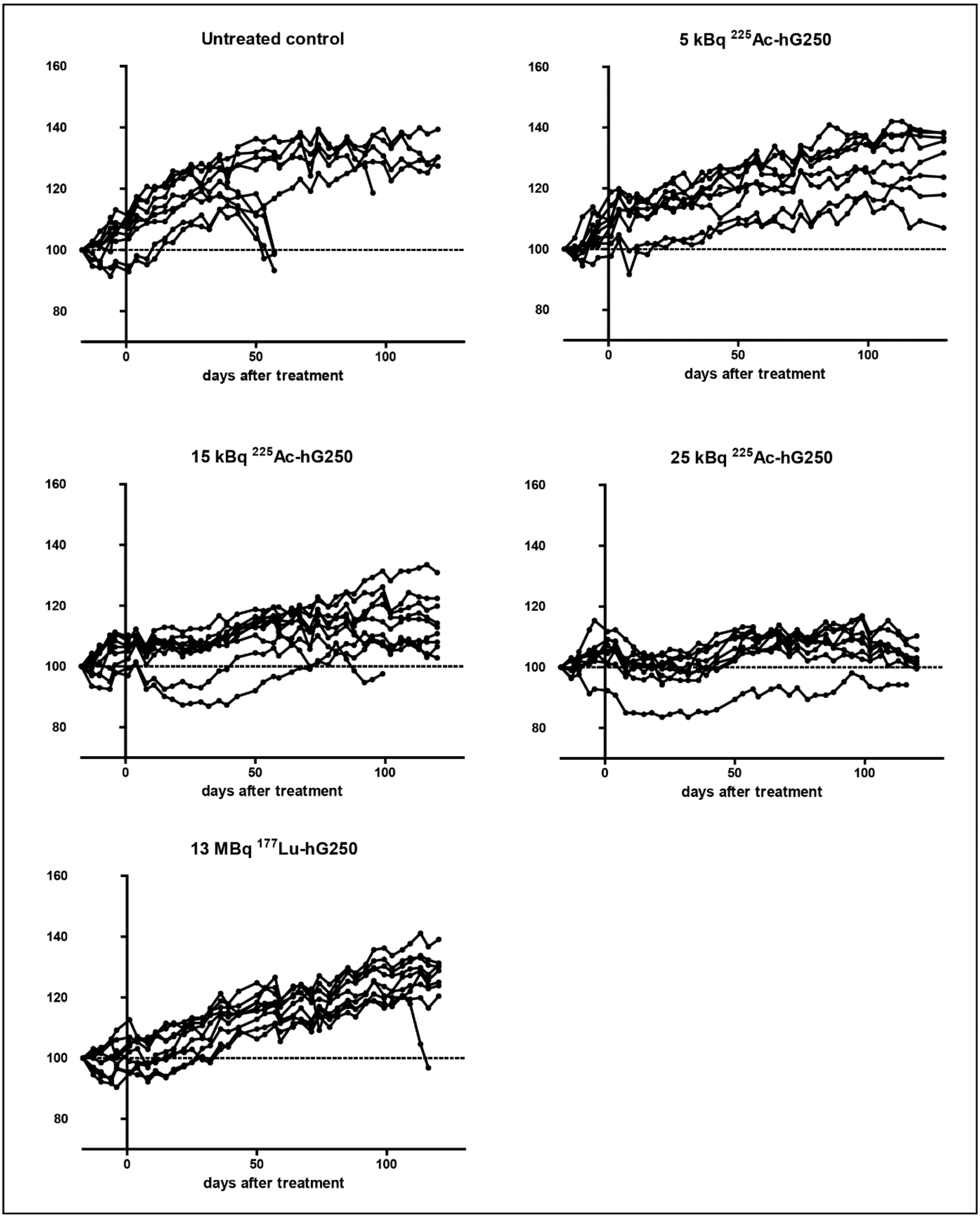

Figure S4. Relative body weight over time in tumor-bearing mice either untreated or treated with different doses of ^{225}Ac-hG250 or ^{177}Lu-hG250.

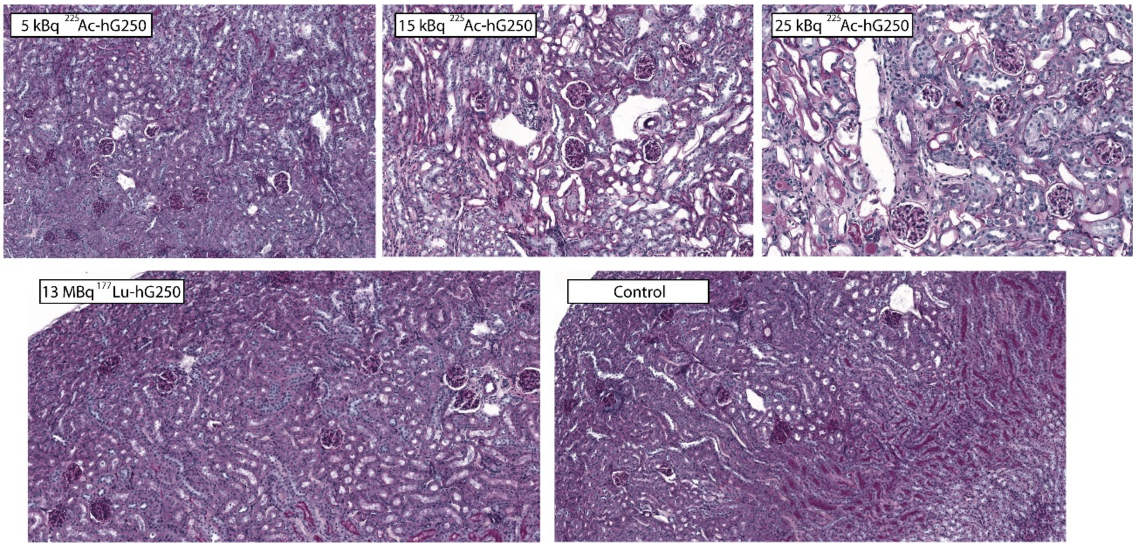

Figure S5. Representative kidney slices stained with periodic acid-Schiff from non-tumor-bearing mice either untreated or treated with different doses of ^{225}Ac-hG250 or ^{177}Lu-hG250, 18 weeks post-treatment. Mice treated with 15 and 25 kBq ^{225}Ac-hG250 show interstitial fibrosis and tubular atrophy in the kidney.
